# Histology-validated comparison of angioscopy, IVUS, and OFDI for assessing stent strut coverage in femoropopliteal arteries with thin neointima

**DOI:** 10.1007/s12928-026-01297-9

**Published:** 2026-05-23

**Authors:** Kazuki Aihara, Sho Torii, Norihito Nakamura, Kaho Hashimoto, Daiki Suzuki, Ryosuke Ohmura, Manabu Shiozaki, Yu Sato, Tsukasa Kato, Yuki Matsumoto, Yuji Ikari, Gaku Nakazawa

**Affiliations:** 1 Department of Cardiology, Tokai University School of Medicine, 143 Shimokasuya, Isehara, 259-1193 Kanagawa Japan; 2 Department of Cardiology, Kindai University, Sakai, Japan

**Keywords:** Angioscopy, Endovascular therapy, Lower extremity artery disease, Drug-Eluting stents, Pathology, Stent healing

## Abstract

In-stent thrombotic occlusion remains a major clinical challenge after femoropopliteal stenting, and delayed healing is believed to contribute to this problem. However, conventional intravascular imaging may struggle to evaluate vascular healing due to limited resolution, particularly in thin neointima. This study aimed to validate the diagnostic performance of angioscopy for assessing endothelial strut coverage in thin neointima using histology as the reference standard. In a healthy swine model, 22 femoropopliteal arteries were evaluated one month after stenting. Ex vivo angioscopy, intravascular ultrasound (IVUS), and optical frequency domain imaging (OFDI) were performed. Endothelial strut coverage was assessed in segments with neointimal thickness < 1000 μm (thin neointima) using histology as the gold standard. Sensitivity, specificity, positive predictive value (PPV), and negative predictive value (NPV) for detecting endothelial-covered struts were calculated. A total of 112 co-registered image sets were obtained, of which 62 were classified as thin neointima. Histology identified endothelial-covered struts in 8 of 62 segments (12.9%). Angioscopy color assessment showed a sensitivity of 100%, specificity of 92%, PPV of 60%, and NPV of 100%. In contrast, IVUS and OFDI demonstrated a high sensitivity for detecting endothelial-covered struts; however, overestimated coverage and failed to distinguish endothelial-covered struts from fibrin-covered struts (sensitivity 100%, specificity 0%, PPV 15%). While IVUS and OFDI overestimate endothelial stent coverage in thin neointima, angioscopy offers superior diagnostic accuracy for identifying fibrin-covered struts and may serve as a valuable modality for assessing vascular healing in femoropopliteal arteries, potentially facilitating the optimization of antithrombotic therapy.

**Graphical abstract:**

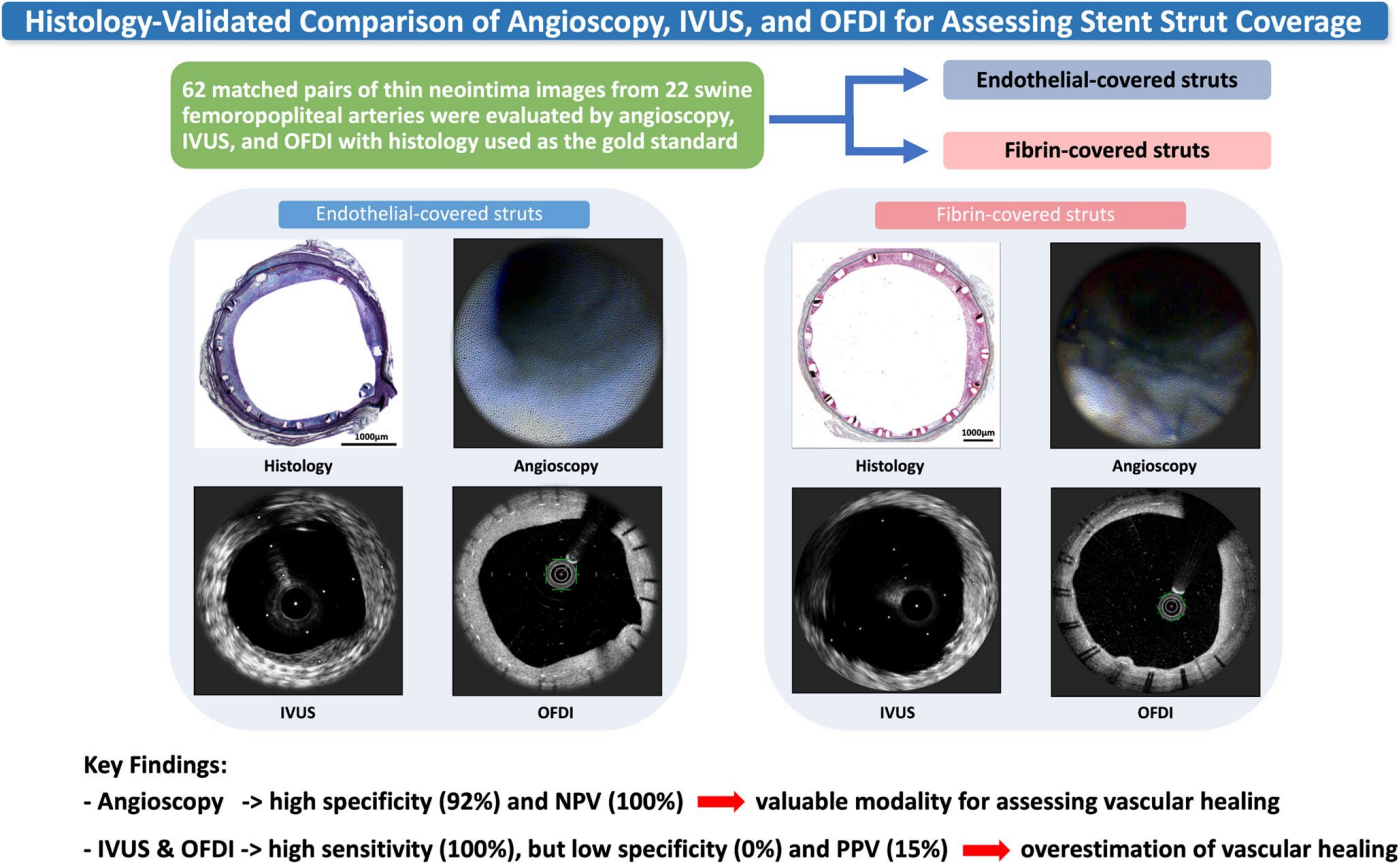

## Introduction

The global prevalence of lower extremity artery disease (LEAD) in adults is estimated at 9.7%, and LEAD is a growing global health concern, leading to a significant increase in both endovascular therapy (EVT) and surgical bypass procedures [1–3]. In recent years, EVT has become the primary treatment strategy for femoropopliteal lesions, and drug-eluting stents (DES) have improved outcomes [4]. However, the long-term clinical outcomes of femoropopliteal stenting, including bare metal stents and DES, remain suboptimal compared to those in coronary artery interventions [5], and in-stent thrombotic occlusion remains a major clinical issue [6]. A previous study has reported that the 1-year restenosis rate after femoropopliteal DES implantation was 12.9%, of which 71% were attributable to in-stent occlusion [7].

A central goal of post-stent vascular healing is complete endothelialization of stent struts. In coronary arteries, incomplete endothelial strut coverage is a well-established substrate for thrombotic complications [8–10], and confirming coverage in vivo could inform antithrombotic strategy. This concern is heightened in the femoropopliteal segment, where delayed vascular healing is believed to contribute to poorer clinical outcomes [11]. Previous case reports have demonstrated that delayed vascular healing after DES implantation has been associated with adverse events such as restenosis and stent thrombosis [12, 13]. Furthermore, prior angioscopic studies have demonstrated that fibrin deposition around the struts is prone to thrombus attachment and may contribute to stent thrombosis [14]. Accordingly, accurate in vivo assessment of endothelialization and fibrin coverage is clinically crucial.

Although intravascular ultrasound (IVUS) and optical frequency domain imaging (OFDI) are widely used, their limited resolution often hinders the precise evaluation of neointimal strut coverage [15]. In contrast, angioscopy offers direct en-face visualization, potentially overcoming these resolution constraints. Previous studies have demonstrated the feasibility and clinical utility of in vivo angioscopy for assessing stent coverage and thrombus formation [16]. However, its diagnostic utility in peripheral arteries remains unestablished. Therefore, the present study aimed to validate the accuracy of angioscopy for assessing strut coverage in thin neointima using histology as the reference standard.

## Methods

### Study framework

This study represents a sub-study of our broader preclinical swine femoropopliteal stent model project, which originally included a total of 18 swine (36 femoropopliteal arteries) [17]. The primary objective of the overarching study was to evaluate the differences in stent behavior among various stent types—including bare nitinol and drug-eluting stents—in a healthy, poor below-the-knee runoff model. From this primary cohort, we conducted two focused imaging-based sub-studies. The first sub-study evaluated the characterization of in-stent restenosis tissue using OFDI, IVUS, and angioscopy in segments with thick neointima (≥ 1000 μm), which included 22 arteries from 11 swine. The second imaging sub-study, which constitutes the current investigation, specifically focused on imaging–histology validation in segments with thin neointima (< 1000 μm), utilizing 22 arteries from 11 swine.

### Animal model and stent implantation procedure

The study protocol received approval from the Japanese Association for Laboratory Animal Science (Approval No. IVT22-90, S23-058). All animals were maintained in accordance with the Animal Welfare Act and Public Health Service guidelines. This preclinical ex vivo validation study employed a healthy swine model to investigate vascular healing following femoropopliteal artery stenting, as previously reported [17]. Briefly, dual antiplatelet therapy was initiated two days prior to the procedure. Under general anesthesia, a 6-French guiding catheter was introduced into the common femoral artery. To reproduce a clinically relevant poor below-the-knee runoff condition—known to be associated with adverse outcomes after femoropopliteal intervention [18]—coils (VortX-18 Diamond coil, Boston Scientific, Marlborough, MA, USA) were deployed in the tibial artery. Thereafter, one of three stent types—bare nitinol stents (Innova; strut thickness about 220 μm; Boston Scientific, Marlborough, MA, USA), fluoropolymer-based paclitaxel-eluting stents (Eluvia; drug dose 0.167 µg/mm², strut thickness about 220 μm; Boston Scientific, Marlborough, MA, USA), or polymer-free paclitaxel-eluting stents (Zilver PTX; drug dose 3.0 µg/mm², strut thickness about 220 μm; Cook Corporation, Bloomington, IN, USA)—was implanted in each femoral artery. All implanted stents were 6.0 × 40 mm in size. All experimental procedures adhered to institutional animal care standards.

### Vessel harvesting and preparation

At one month, follow-up angiography was performed to evaluate the vascular state. The swine were then euthanized with intravenous pentobarbital and/or potassium. Stented vessels were excised, pressure-perfused with 0.9% saline to remove residual blood, and subsequently fixed by perfusion with 10% formalin. Post-fixation radiography confirmed complete stent expansion without evidence of fracture.

### Ex vivo intravascular imaging acquisition

After formalin fixation, the stented arteries were imaged ex vivo while immersed in a 0.9% saline bath. Imaging was performed from the distal to the proximal segments using angioscopy (VISIBLE; FiberTech, Chiba, Japan), IVUS (AltaView, Terumo, Tokyo, Japan), and OFDI (LUNAWAVE, Terumo, Tokyo, Japan). IVUS images were acquired at a pullback rate of 0.5 mm/s, and OFDI images at 20 mm/s [19]. All imaging data were analyzed at our institution. The stented segments were evaluated at 1-mm intervals. Angioscopic assessment evaluated both neointimal color and coverage grade. Neointimal color was classified as either white or red [20]; struts covered by white neointima in angioscopy were defined as endothelial-covered struts, whereas those surrounded by red neointima were defined as fibrin-covered struts. Additionally, neointimal coverage was graded using a 4-point scale [14]: grade 0 (struts fully visible); grade 1 (struts bulging but translucently visible); grade 2 (struts embedded but translucently visible); and grade 3 (struts fully embedded and invisible). In this study, only grade 3 was defined as endothelial-covered struts. For IVUS and OFDI, struts were defined as covered if any identifiable tissue signal (> 0 μm) was visible overlying the struts. This 0 μm threshold was chosen to reflect previous clinical studies, where any detectable tissue layer is often interpreted as an indicator of the coverage, regardless of its thickness [15, 21].

### Histological processing and definition of strut coverage

**Fig. 1:**
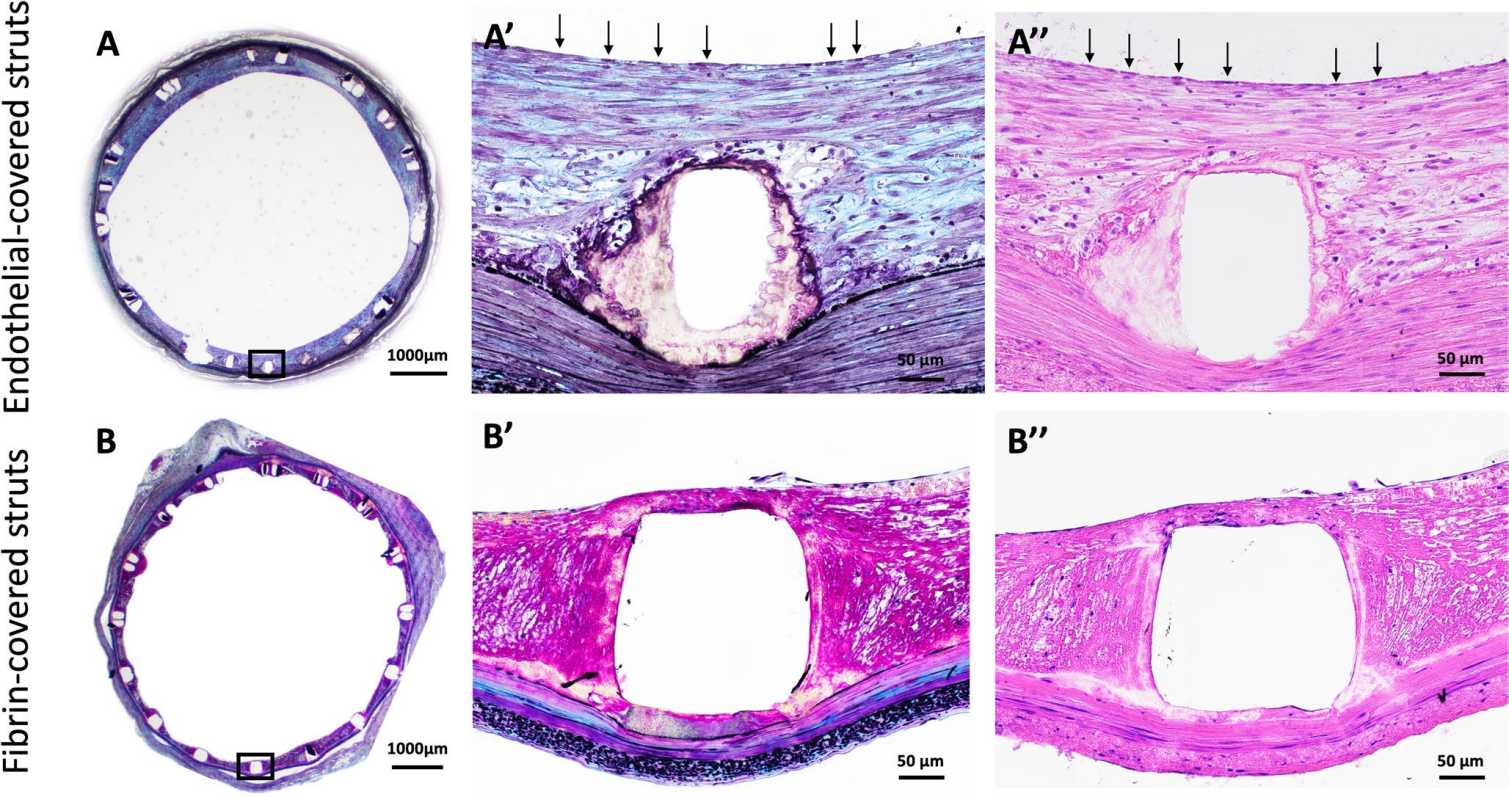
Definition of strut coverage. Representative photomicrographs of endothelial-covered struts (**A**) and fibrin-covered struts (**B**) stained with Movat Pentachrome. Endothelial-covered struts were defined as the presence of luminal endothelial cells overlying struts and an underlying smooth muscle cell layer. High-power Movat Pentachrome and hematoxylin and eosin staining images show endothelial-covered (A’ and A’’) and fibrin-covered struts (B’ and B’’). Arrows indicate endothelial cells

Following imaging, stented arteries were embedded in methyl methacrylate resin. The stented segments were cut at 3 mm intervals, and 4–6 μm thick sections were prepared and stained with hematoxylin and eosin and Movat Pentachrome for light microscopic evaluation [17, 22, 23]. Although stents were 40 mm in length and sectioned at 3 mm intervals, not all sections were suitable for final analysis. Sections with significant processing artifacts or inadequate tissue preservation were excluded prior to image co-registration. Neointimal coverage of each strut was classified as either endothelial-covered or fibrin-covered struts according to the following definition. Endothelial coverage was defined as the presence of luminal endothelial cells overlying struts and an underlying smooth muscle cell layer (Fig. 1) [15, 24]. Struts covered only by fibrin thrombus were considered fibrin-covered. Each segment was defined as endothelial coverage if more than 90% of the struts were endothelial-covered.

### Image and histology co-registration

**Fig. 2:**
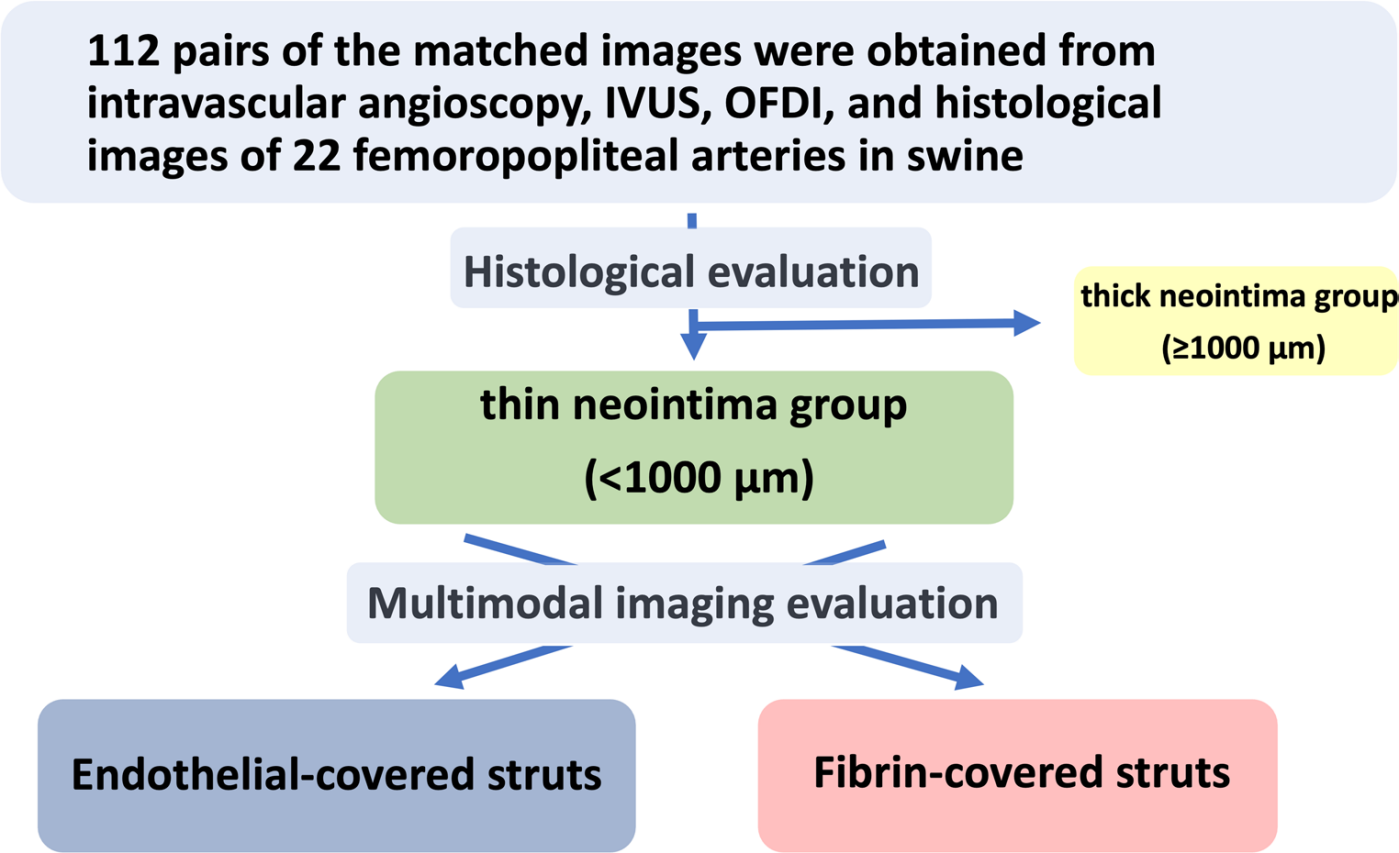
Lesion flowchart for the current study. From 22 swine femoropopliteal arteries, 112 matched images were evaluated. Segments were classified by neointimal thickness: thin (< 1000 μm) vs. thick (≥ 1000 μm). Thin neointima segments were evaluated by angioscopy, IVUS, and OFDI. IVUS: intravascular ultrasound; OFDI: optical frequency domain imaging

Imaging frames from angioscopy, IVUS, and OFDI were co-registered to histological sections using anatomic landmarks and measured distances from stent edges. The analysis focused exclusively on segments where the neointimal thickness was less than 1000 μm based on histological evaluation, which were defined as the “thin neointima group” (Fig. 2). Neointimal thickness was defined as the maximum neointimal thickness measured within each histological section, rather than an average thickness across the segment. The predefined cutoff of 1000 μm was therefore applied to the maximum measured thickness per segment. Only segments that fulfilled all the following predefined criteria were included in the final analysis: [1] adequate histological quality without sectioning artifacts [2], successful co-registration across angioscopy, IVUS, OFDI, and histology, and [3] histologically confirmed neointimal thickness < 1000 μm. The primary diagnostic accuracy assessment was subsequently performed on the thin neointima group. Two experienced investigators (N.N. and S.T.), who were blinded to the histological findings, independently evaluated all images to determine coverage status for each modality.

### Statistical analysis

Using histological findings as the gold standard, the diagnostic accuracy of each imaging modality for distinguishing endothelial-covered struts was assessed by calculating sensitivity, specificity, positive predictive value (PPV), and negative predictive value (NPV) at the segment level. To address the potential within-animal correlation among multiple histological sections, all diagnostic metrics (sensitivity, specificity, PPV, and NPV) were first summarized on a per-animal basis. Each animal was treated as an independent observational unit, and the mean values of each metric were calculated across animals. To estimate the variability and 95% confidence intervals (CI) of these metrics while considering within-animal clustering, we performed cluster bootstrap resampling (2,000 iterations) at the animal level. This method appropriately accounts for the non-independence of observations from the same animals and provides a valid CI for diagnostic performance. Inter-observer agreement between two independent investigators was evaluated for each imaging modality. Cohen’s kappa coefficient was calculated when applicable, and percent agreement was reported when kappa could not be reliably estimated due to limited variability in classification. All statistical analyses were performed using JMP software version Student Edition 18.0 (SAS Institute, Cary, North Carolina, USA) and Python software version 1.10.1 (SciPy library; Python Software Foundation, Wilmington, Delaware, USA).

## Results

### Baseline animal and angiographic outcomes

All swine completed the scheduled one-month observation period without significant adverse events. Follow-up angiography demonstrated no evidence of vessel dissection, ectasia, or aneurysm. Post-fixation radiographs of the stented arteries confirmed complete stent expansion without fractures. Furthermore, all tibial arteries subjected to coil embolization were fully occluded, accompanied by the development of bridging collateral vessels supplying the affected regions.

### Histological findings and cohort description

A total of 36 femoropopliteal arteries from 18 swine were included in the experimental model. Of these, 112 co-registered sections from 22 arteries in 11 swine fulfilled the predefined criteria—adequate histological quality and successful multimodality co-registration—and were eligible for analysis. Based on maximum neointimal thickness per histological section, 62 sections were classified as thin neointima (< 1000 μm) and included in the primary analysis, whereas 50 Sect.  (44.6%) were categorized as thick neointima (≥ 1000 μm). Within the thin neointima group, the median neointimal thickness was 57 μm (IQR 27–247 μm; range 15–824 μm). Histological evaluation identified 8 of the 62 segments (12.9%) with endothelial-covered struts (Table 1).

**Table 1:** Actual interpretation of neointimal coverage by angioscopy, IVUS, OFDI, and histology in the thin neointima group

Thin neointima group		Histology (*n*=62)	
		Endothelial-covered struts	Fibrin-covered struts
Angioscopic color category	Endothelial-covered struts	8	6
	Fibrin-covered struts	0	48
Angioscopic coverage grade (=3)	Endothelial-covered struts	6	6
	Fibrin-covered struts	2	48
IVUS	Endothelial-covered struts	8	54
	Fibrin-covered struts	0	0
OFDI	Endothelial-covered struts	8	54
	Fibrin-covered struts	0	0

IVUS = intravascular ultrasound; OFDI = optical frequency domain imaging

### Diagnostic accuracy in thin neointima (< 1000 μm)

**Fig. 3:**
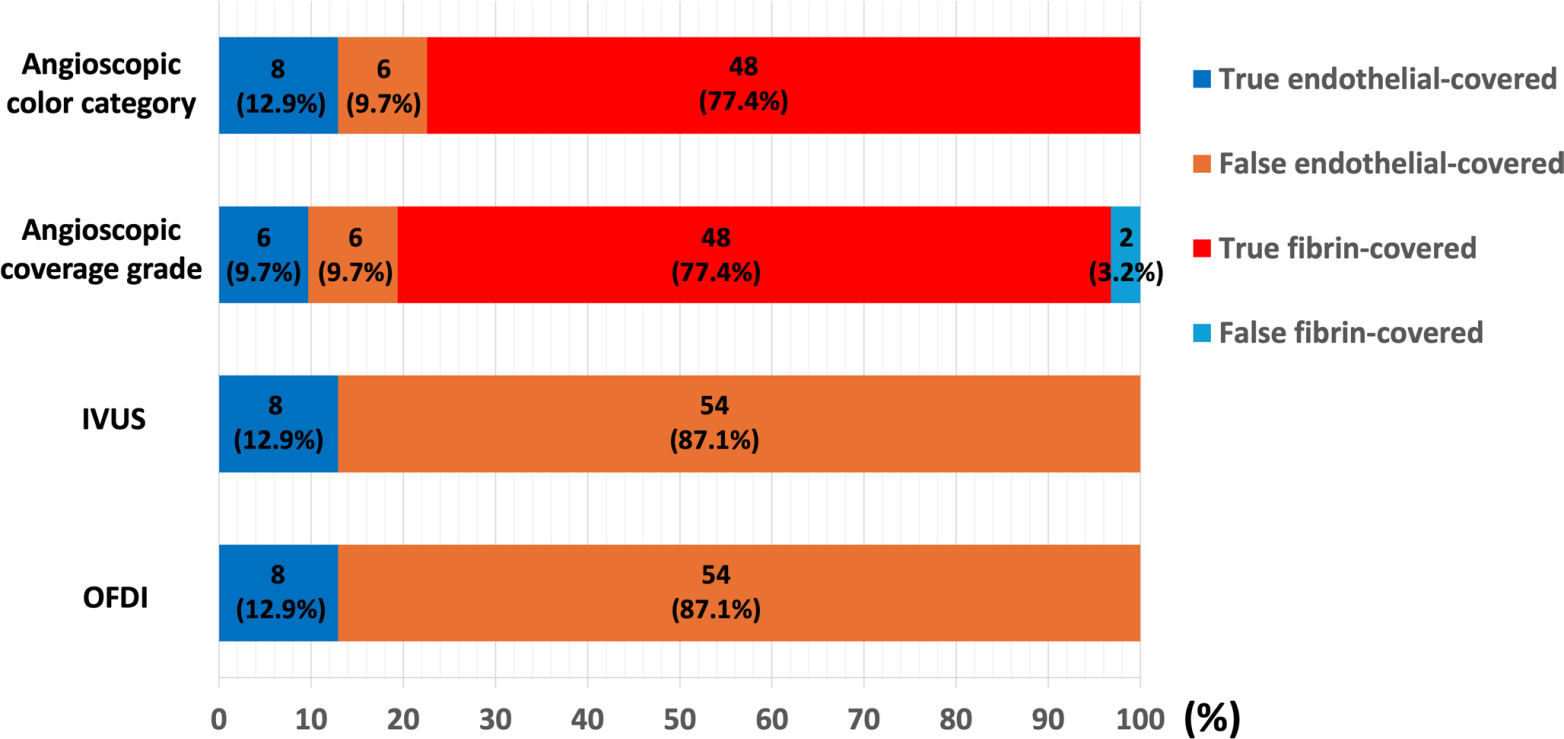
Actual interpretation of neointimal coverage by angioscopy, IVUS, OFDI, and histology in the thin neointima group. Stacked bar charts show the distribution of diagnostic classifications for each imaging modality compared with histology as the reference in segments with thin neointima (< 1000 μm). Categories include true and false endothelial-covered and fibrin-covered struts. Values represent counts and percentages. IVUS: intravascular ultrasound; OFDI: optical frequency domain imaging

**Fig. 4:**
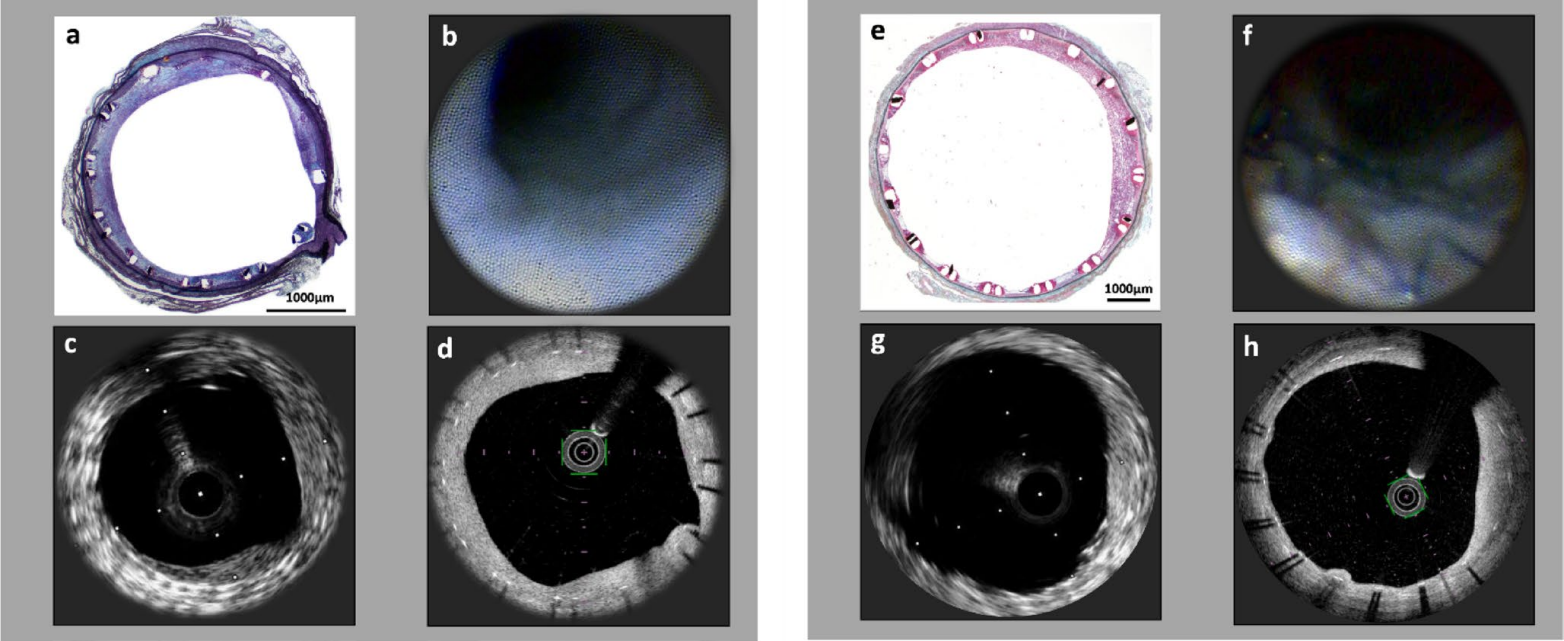
Representative co-registered images in the thin neointima group. (**A**) Endothelial-covered struts: (**a**) Histology (Movat Pentachrome) shows extracellular matrix neointima. (**b**) Angioscopy shows a white surface; struts are invisible. (**c**) IVUS shows isoechoic neointima. (**d**) OFDI shows homogenous backscattering. (**B**) Fibrin-covered struts: (**e**) Histology shows fibrin surrounding struts. (**f**) Angioscopy shows a partially red surface. (**g**) IVUS and (**h**) OFDI show appearances similar to endothelial-covered struts, highlighting the difficulty in differentiation. IVUS: intravascular ultrasound; OFDI: optical frequency domain imaging

**Fig. 5:**
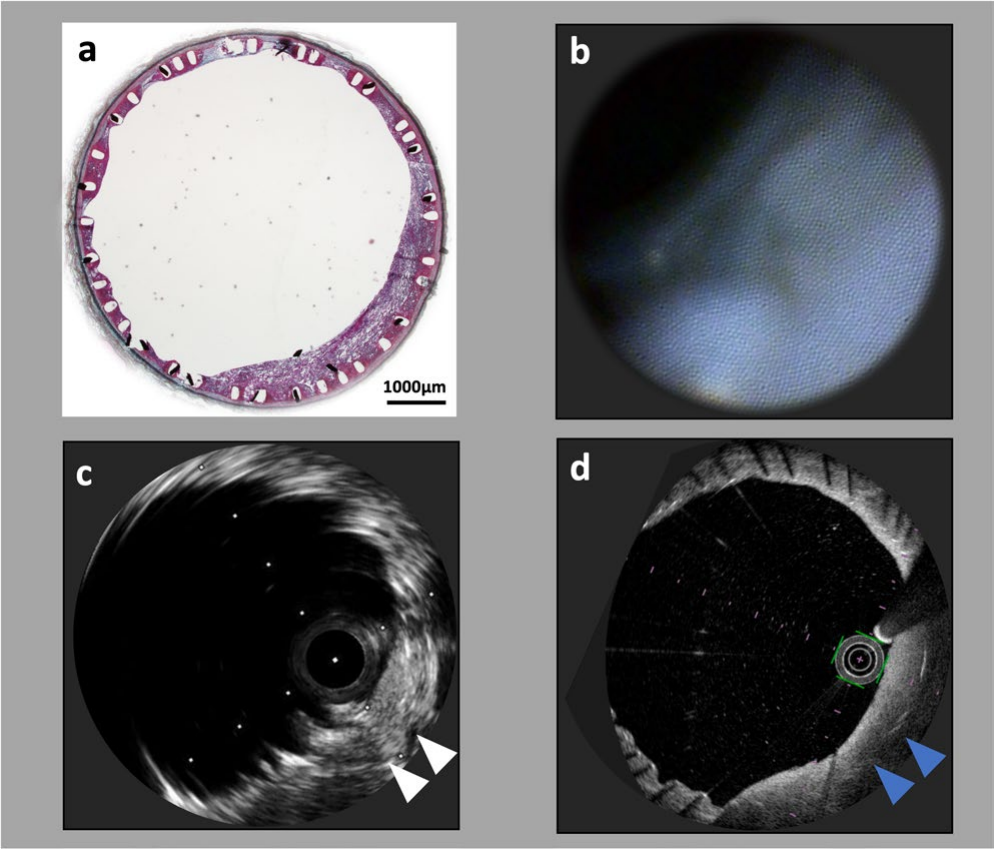
Overestimation of endothelial-covered struts on angioscopy. (**a**) Histology shows fibrin around struts (fibrin-covered struts). (**b**) Angioscopy shows a white surface, mimicking endothelial coverage (false positive). (**c**, **d**) IVUS and OFDI also show tissue coverage signals (white and blue arrowheads). IVUS: intravascular ultrasound; OFDI: optical frequency domain imaging

Using histology as the gold standard, the diagnostic performance of each imaging modality for detecting endothelial-covered stent struts was assessed in the thin neointima (Tables 1 and 2; Figs. 3 and 4). Inter-observer agreement was assessed prior to diagnostic accuracy analysis. For IVUS and OFDI, kappa could not be reliably estimated because one observer classified nearly all segments into a single category; percent agreement was 100% and 98.4%, respectively. Angioscopy demonstrated substantial agreement (κ = 0.83, 95% CI: 0.66–0.99). Angioscopic assessment based on neointimal color accurately detected endothelial-covered segments, with a sensitivity of 100% (95% CI: 100–100), a specificity of 92% (95% CI: 79.9–100), a PPV of 60% (95% CI: 0.0–100), and an NPV of 100% (95% CI: 100–100). Assessment based on coverage grades identified 13 segments (21.0%) as grade 3, showing acceptable diagnostic performance: sensitivity 67% (95% CI: 20.0–100), specificity 84% (95% CI: 66.7–97.2), PPV 49% (95% CI: 16.7–85.7), and NPV 95% (95% CI: 87.1–100). However, the PPV for both color and coverage grade assessments was approximately 50–60%, suggesting that angioscopy tended to overestimate endothelial coverage—particularly in segments with thick, fibrin-rich neointima—resulting in false positives (Fig. 5). In contrast, the diagnostic utility of IVUS and OFDI was limited in this cohort. Both modalities showed a sensitivity of 100% and a specificity of 0%, with a PPV of 15% and an undefined NPV (Table 3). These modalities consistently overestimated endothelial coverage in thin neointima, misclassifying fibrin coverage as endothelial coverage.

**Table 2:** Assessment of angioscopic coverage grade in the thin neointima group

Angioscopic coverage grade (%)			
Grade 0	Grade 1	Grade 2	Grade 3
0 (0)	25 (40.3)	24 (38.7)	13 (21.0)

**Table 3:** Accuracy of angioscopy, IVUS, and OFDI for assessing endothelial-covered stent struts in the thin neointima group

Thin neointima group				
	Sensitivity	Specificity	PPV	NPV
Angioscopic color category (%)	100 (CI: 100–100)	92 (CI: 79.9–100)	60 (CI: 0.0–100)	100 (CI: 100–100)
Angioscopic coverage grade (=3) (%)	67 (CI: 20.0–100)	84 (CI: 66.7–97.2)	49 (CI: 16.7–85.7)	95 (CI: 87.1–100)
IVUS (%)	100 (CI: 100–100)	0 (CI: 0.0–0.0)	15 (CI: 3.3–28.8)	N/A
OFDI (%)	100 (CI: 100–100)	0 (CI: 0.0–0.0)	15 (CI: 3.3–28.8)	N/A

CI = confidence interval; IVUS = intravascular ultrasound; N/A = not applicable; NPV = negative predictive value; OFDI = optical frequency domain imaging; PPV = positive predictive value

## Discussion

This study provides novel, histology-validated evidence that angioscopy may serve as a complementary modality to identify endothelial-covered struts within its visual field, particularly when conventional imaging yields ambiguous results in a swine femoropopliteal stent model, specifically within thin neointima. A key finding of this study is that conventional imaging modalities (IVUS and OFDI) completely failed to detect fibrin coverage in thin neointima (0% specificity), whereas angioscopy demonstrated high specificity and negative predictive value.

As part of our overall study design, we stratified segments according to neointimal thickness. In a separate analysis, we focused on segments with neointimal thickness ≥ 1000 μm and demonstrated that both IVUS and OFDI showed acceptable performance for tissue characterization in thick neointima. In contrast, the present study specifically targeted segments with neointimal thickness < 1000 μm, a condition in which tissue characterization is inherently more challenging. By focusing on thinner neointima, we sought to determine the diagnostic accuracy and limitations of imaging modalities, including angioscopy, for assessing stent strut coverage, given that incomplete coverage represents a key substrate for stent thrombosis. Although neointimal thickness may be an important predictor of endothelial coverage, the present study was designed to focus on the diagnostic accuracy of each imaging modality in thin neointima rather than thickness-based evaluation.

The performance discrepancies among modalities reflect their underlying imaging principles. Notably, there were no significant differences in sensitivity, specificity, PPV, or NPV between IVUS and OFDI, indicating comparable diagnostic limitations in the thin neointima setting. Although IVUS and OFDI offer high resolution, their ability to characterize the neointimal tissue types is limited when neointimal tissue is minimal [15]. In this study, both modalities consistently overestimated vascular healing. This is likely because thin fibrin deposition on stent struts does not produce sufficient attenuation or low-intensity signals that allow neointimal tissue characteristics to be distinguished, leading to the misclassification of fibrin coverage as endothelial coverage. This mechanism explains the critically low specificity observed with these modalities.

In contrast, angioscopy demonstrated superior diagnostic accuracy by providing direct visualization of the luminal surface. Typically, endothelial coverage appeared white, while fibrin coverage appeared red. Consequently, angioscopy could reliably rule out fibrin coverage (NPV 100%), a capability that IVUS and OFDI lacked. However, the results also highlight a limitation: the PPV was modest (~ 60%). This suggests that while angioscopy is excellent at identifying “danger” (fibrin-covered struts), a “white” appearance may still conceal incomplete endothelialization due to fibrin coverage. Clinicians should be aware that a white angioscopic appearance does not guarantee complete histological endothelialization, although it is significantly more reliable than IVUS/OFDI findings.

In coronary intervention, endothelial strut coverage is a surrogate marker for vascular healing and thrombotic risk [9]. In the femoropopliteal artery, where identifying the optimal duration of antithrombotic therapy remains a challenge, precise assessment of coverage is equally important [7, 11, 12]. Given that IVUS and OFDI tend to erroneously confirm endothelial coverage in thin neointima, relying solely on these modalities could lead to premature discontinuation of antithrombotic therapy. Angioscopy can mitigate this risk by identifying fibrin-covered struts that are invisible to other modalities.

At present, angioscopy is not routinely recommended in major international guidelines for post-stent evaluation, and its clinical use remains limited. One of its principal weaknesses is the restricted field of view, which prevents full circumferential assessment of the vessel wall. Therefore, angioscopy should not be considered a universal imaging strategy for all stented lesions. Rather, it may have a potential role in carefully selected clinical scenarios—for example, in patients at high bleeding risk in whom early de-escalation of dual antiplatelet therapy is being contemplated. In such situations, confirmation of vascular healing by angioscopy might provide supplementary information. Conversely, if persistent fibrin coverage is observed, continuation of dual antiplatelet therapy or careful consideration of additional anticoagulant treatment could be entertained on an individualized basis. These potential applications require further prospective evaluation before routine clinical implementation can be recommended.

### Study limitations

This study has several limitations. First, the experiment utilized healthy swine arteries. While this model is well-established for stent evaluation, it lacks the complex atherosclerotic plaque burden and calcification typical of human femoropopliteal disease; thus, the histological features of the neointima may not fully replicate those seen in clinical practice. Second, imaging was performed ex vivo without physiological blood flow. Although this precludes the assessment of hemodynamic influences and may differ from in vivo findings, the ex vivo design was essential to ensure precise co-registration between imaging modalities and histology, which is virtually impossible in a clinical setting. In addition, adequate blood clearance—required for both angioscopy and OFDI—is technically challenging in the high-flow femoropopliteal model in vivo and may introduce variability in image interpretation. The ex vivo approach allowed standardized visualization across modalities and minimized procedural artifacts, including potential disruption of delicate fibrin deposition during catheter manipulation, thereby enabling a more controlled assessment of stent strut coverage. Third, the sample size was relatively small (22 arteries from 11 swine), and the absence of a pre-specified power calculation constitutes a limitation of this study. Nevertheless, this number is comparable to previous preclinical validation studies [17, 25], and we employed cluster bootstrap resampling to account for within-animal correlations, thereby ensuring statistical robustness. Fourth, the diagnostic assessment of angioscopy is limited by its forward-looking field of view. Unlike IVUS or OFDI, which provide 360-degree cross-sectional images, angioscopy may fail to evaluate struts located in certain quadrants or behind curves. Therefore, its high NPV observed in this study should be interpreted within the context of the visualized luminal surface, and it should be viewed as a tool to supplement, rather than replace, cross-sectional imaging. Finally, although identifying a thickness-based cutoff to predict endothelial coverage may be clinically meaningful, this was not the primary objective of the present study, which focused on the diagnostic accuracy of each imaging modality. In addition, given the relatively small sample size, the present dataset may be underpowered to establish a reliable thickness-based cutoff; therefore, thickness-based analyses such as ROC curve assessment were not included.

## Conclusions

While IVUS and OFDI tend to overestimate endothelial stent coverage in thin neointima, angioscopy offers superior diagnostic accuracy for identifying fibrin-covered struts. These findings indicate that angioscopy may serve as a valuable modality for assessing vascular healing in femoropopliteal arteries, potentially facilitating the optimization of antithrombotic therapy.

## Abbreviations

DES: Drug-eluting stent
EVT: Endovascular therapy
IVUS: Intravascular ultrasound
LEAD: Lower extremity artery disease
NPV: Negative predictive value
OFDI: Optical frequency domain imaging
PPV: Positive predictive value
